# GBP2 is a prognostic biomarker and associated with immunotherapeutic responses in gastric cancer

**DOI:** 10.1186/s12885-023-11308-0

**Published:** 2023-10-02

**Authors:** Yunfei Wang, Jiadong Pan, Fangmei An, Ke Chen, Jiawei Chen, He Nie, Yanping Zhu, Zhengtao Qian, Qiang Zhan

**Affiliations:** 1 Departments of Gastroenterology, Wuxi People’s Hospital, Wuxi Medical Center, The Affiliated Wuxi People’s Hospital of Nanjing Medical University, Nanjing Medical University, Wuxi, Jiangsu 214023 China; 2 Departments of Gastroenterology, The Third People’s Hospital of Kunshan, Suzhou, 215300 China; 3 Department of Clinical Laboratory, Changshu Medicine Examination Institute, Changshu, 215500 China

**Keywords:** GBP2, Gastric cancer, Immunotherapy, Biomarker

## Abstract

**Background:**

The interferon-induced protein known as guanylate-binding protein 2 (GBP2) has been linked to multiple different cancer types as an oncogenic gene. Although the role of GBP2 in cancer has been preliminarily explored, it is unclear how this protein interacts with tumor immunity in gastric cancer.

**Methods:**

The expression, prognostic value, immune-correlations of GBP2 in gastric cancer was explored in multiple public and in-house cohorts. In addition, the pan-cancer analysis was performed to investigate the immunological role of GBP2 based on The Cancer Genome Atlas (TCGA) dataset, and the predictive value of GBP2 for immunotherapy was also examined in multiple public cohorts.

**Results:**

GBP2 was highly expressed in tumor tissues and associated with poor prognosis in gastric cancer. In addition, GBP2 was associated with the immune-hot phenotype. To be more specific, GBP2 was positively related to immuno-modulators, tumor-infiltrating immune cells (TIICs), immunotherapy biomarkers, and even well immunotherapeutic response. In addition to gastric cancer, GBP2 was expected to be an indicator of high immunogenicity in most cancer types. Importantly, GBP2 could predict the immunotherapeutic responses in at least four different cancer types, including melanoma, urothelial carcinoma, non-small cell lung cancer, and breast cancer.

**Conclusions:**

To sum up, GBP2 expression is a promising pan-cancer biomarker for estimating the immunological characteristics of tumors and may be utilized to detect immuno-hot tumors in gastric cancer.

**Supplementary Information:**

The online version contains supplementary material available at 10.1186/s12885-023-11308-0.

## Background

The tumor immune microenvironment (TIME) is a dynamic network structure with high heterogeneity, consisting of diverse immune cells, fibroblast cells, vascular endothelial cells, together with extracellular matrix (ECM) and numerous cytokines [1]. It plays a role as huge as solid malignant tissues during tumor progression [2]. TIME participate broadly in the acquisition and maintenance of the earmarks of cancer, such as sustaining proliferative signaling, resisting tumor cell death, inducing angiogenesis, activating invasion and metastasis, etc., which offers a therapeutic target in cancer [3, 4]. The targets include but are not limited to non-cancerous cells and components presented in the tumor. Importantly, TIME determines the efficacy of multiple treatments, including immunotherapy, chemotherapy, and radiotherapy [1, 5].

Cancer immunotherapy-developed on the basis of tumor escape mechanism reactivates anti-tumor response by manipulating the immune system and restrains the pathways leading to escape [6]. As a part of cancer immunotherapies, immune checkpoint blockade (ICB) has come to the foreground in recent years, including cytotoxic T lymphocyte-associated protein 4 (CTLA-4) inhibitor, programmed death protein 1 (PD-1) inhibitor, and programmed cell death 1 ligand 1 (PD-L1) inhibitor, which are widely applied to the treatment of renal cell carcinoma, lung cancer, and gastric cancer, etc. [7]. Although ICB has been deemed to revolutionize tumor treatability, several problems are still needed to be addressed, such as lack of persistence in a minority of patients and severe toxic responses [8]. Therefore, novel immune hallmarks are needed to be found to complement cancer immunotherapy in order to make it more effective and reliable.

Guanylate-binding proteins (GBPs), assembled by interferon (IFN)-induced GTPases, serve as a major nexus in cell-autonomous immunity against microbial pathogens, inflammation and cancer [9]. Abnormal expression of GBPs is usually observed in various tumors and play significant roles in oncogenesis and tumor progression [10–12]. Remarkably, Godoy et al. found that GBP2 was overexpressed in breast cancer and significantly associated with better prognosis, and also indicated efficient T cell response [13]. Wang et al. discovered that high GBP2 expression in proficient-mismatch-repair or microsatellite stability (pMMR/MSS) colorectal cancer patients may have better efficacy of anti-PD-1 therapy [14]. However, it still remains unclear whether GBP2 could act as biomarker in other cancers.

In the current, we first analyzed the expression and immuno-correlations of GBP2 in gastric cancer and expand its immuno-related role in pan-cancer. GBP2 was upregulated in tumor tissues but associated with well prognosis. In addition, GBP2 was positively correlated with activated TIME features and well immunotherapeutic responses. Furthermore, pan-cancer analysis revealed that GBP2 was associated with activated TIME features in most cancer types. Overall, this study summarized the immuno-correlations of GBP2 in gastric cancer and pan-cancer, and GBP2 could be a novel biomarker for the predication of immunotherapeutic efficacy in most cancers.

## Methods

### Acquisition of public data

The pan-cancer (TOIL RSEM tpm) and gastric cancer (IlluminaHiSeq) RNA-sequencing (RNA-seq) data as well as clinical annotations of The Cancer Genome Atlas (TCGA) dataset were obtained from the UCSC Xena website (https://xenabrowser.net/datapages/). The abbreviations for various cancer types are exhibited in Supplementary Table S1. Four public datasets comprising RNA-seq data from patients receiving immunotherapy were downloaded from the Gene Expression Omnibus (GEO, http://www.ncbi.nlm.nih.gov/geo/) or the Tumor Immune Dysfunction and Exclusion (TIDE, http://tide.dfci.harvard.edu/) databases, including PRJEB25780 (a prospective phase 2 clinical trial of patients with gastric cancer who are treated with pembrolizumab as salvage treatment) [15], PRJEB23709 (a retrospective cohort of melanoma patients treated with anti-PD-1 monotherapy or combined anti-PD-1 and anti-CTLA-4) [16], GSE176307 (a retrospective cohort of urothelial cancer patients treated with at least one dose of anti-PD-1 or anti-PD-L1 monotherapy) [17], GSE126044 (a retrospective cohort of non-small cell lung cancer patients treated with either nivolumab or pembrolizumab) [18], and MEDI4736 (a single arm neoadjuvant clinical trial of triple negative breast cancer patients receiving durvalumab concurrent with weekly nab-paclitaxel ×12 weeks followed by durvalumab plus dose dense doxorubicin/cyclophosphamide ×4 weeks) [19] cohorts.

### Enrichment analysis of GBP2 in gastric cancer

To identify GBP2-related functions in gastric cancer, we first extracted correlated genes with GBP2 in the TCGA dataset using the LinkedOmics tool [20]. Then, the top 50 positively and negatively correlated genes were submitted for enrichment analysis. Briefly, The h.all.v7.4.symbols.gmt was downloaded from the Molecular Signatures Database (http://www.gsea-msigdb.org/gsea/downloads.jsp) [21] and used for enrichment analysis in the term of Hallmark gene sets. The latest gene annotation of KEGG pathway was obtained (https://www.kegg.jp/kegg/rest/keggapi.html) and was used as background [22–24]. Then, the enrichment analysis was performed using the R package clusterprofiler (version 3.14.3) to obtain the results of gene set enrichment in the term of KEGG analysis. The minimum gene set was set to 5, the maximum gene was set to 5000 and one thousand resamplings was set. P value < 0.05 was considered statistically significant.

### Immunological correlation of GBP2 in gastric cancer

The immunological characteristics of TIME in gastric cancer contained immunomodulators, tumor purity, infiltration levels of tumor-infiltrating immune cells (TIICs), and the expression of immune checkpoints [25]. Firstly, we studied the expression of 150 immunomodulators, including MHC, receptors, chemokines, immunoinhibitors, and immunostimulators [26]. In addition, the correlations between GBP2 and immune checkpoints levels were evaluated. Moreover, the TIMER algorithm [27] was used to estimate TIICs abundance and the correlation between GBP2 and TIICs was also assessed. To investigate the associations between GBP2 and anti-tumor immunity in gastric cancer, the correlations between GBP2 and immunological characteristics of TIME were assessed.

### Correlation between GBP2 and immunotherapeutic response

According to a previous report, immunophenoscore (IPS) was calculated to predict therapeutic response to immunotherapy [28]. The IPS values of gastric cancer patients were obtained from the Cancer Immunome Atlas (TCIA) website (http://tcia.at/home/). In addition, the correlation between GBP2 and four mismatch repair (MMR) genes (MLH1, MSH2, MSH6, and PMS2) were also assessed [29].

### Pan-cancer analysis of immunological correlation of GBP2

To evaluate the pan-cancer immunological correlation of GBP2, we collected pan-cancer expression of 150 immunomodulators, including MHC, receptors, chemokines, immunoinhibitors, and immunostimulators. Then, the correlations between GBP2 and tumor purity as well as TIICs abundance were also assessed. The pan-cancer analysis was conducted using the Sangerbox tool [30].

### Clinical samples

The gastric cancer tissue microarray (TMA, Cat. HStmA180Su19) was purchased from Outdo BioTech (Shanghai, China). A total of 94 tumor samples and 86 para-tumor samples were contained in this research. The clinic-pathological and follow-up data were provided by Outdo BioTech as well. Ethical approval for the use of TMAs was granted by the Clinical Research Ethics Committee in Outdo Biotech.

### Immunohistochemistry (IHC) staining and semi-quantitative scoring

IHC staining was conducted on the above sections according to the standardized procedures. Sections were retrieved by EDTA. The primary antibodies used were as follows: antiGBP2 (1:3000 dilution, Cat. 11854-1-AP, ProteinTech), antiPD-L1 (Ready-to-use, Cat. GT2280, GeneTech), antiPD-1 (Ready-to-use, Cat. GT2281, GeneTech), antiCD8 (Ready-to-use, Cat. GT2112, GeneTech), antiMLH1 (Ready-to-use, Cat. GT2304, GeneTech), antiMSH2 (Ready-to-use, Cat. GT2310, GeneTech), antiMSH6 (Ready-to-use, Cat. GT2195, GeneTech), and antiPMS2 (Ready-to-use, Cat. GT2149, GeneTech). Staining was visualized with DAB and hematoxylin counterstain, and stained sections were captured using Aperio Digital Pathology Slide Scanners. For semi-quantitative analysis, the stained sections were independently evaluated by two pathologists. GBP2 and PD-L1 were assessed by according to the evaluation standard on a 12-point scale by calculating the immunoreactivity score (IRS) [31]. CD8 and PD-1 were assessed by estimating the percentage of cells with strong intensity of membrane staining in the stromal cells.

### Statistical analysis

All statistical analyses were conducted using SPSS 26.0 software or R language. All data are presented as means ± SDs. The difference between the two groups was analyzed by parametric Student’s t-test or non-parametric Mann Whitney test. Survival analysis was performed by log-rank test. Correlation analysis between two variables was analyzed by Pearson test. All statistical tests were two-sided, and P value < 0.05 was considered statistically significant and labeled with *P < 0.05; **P < 0.01; ***P < 0.001.

## Results

### Expression and prognostic value of GBP2 in gastric cancer

**Fig. 1:**
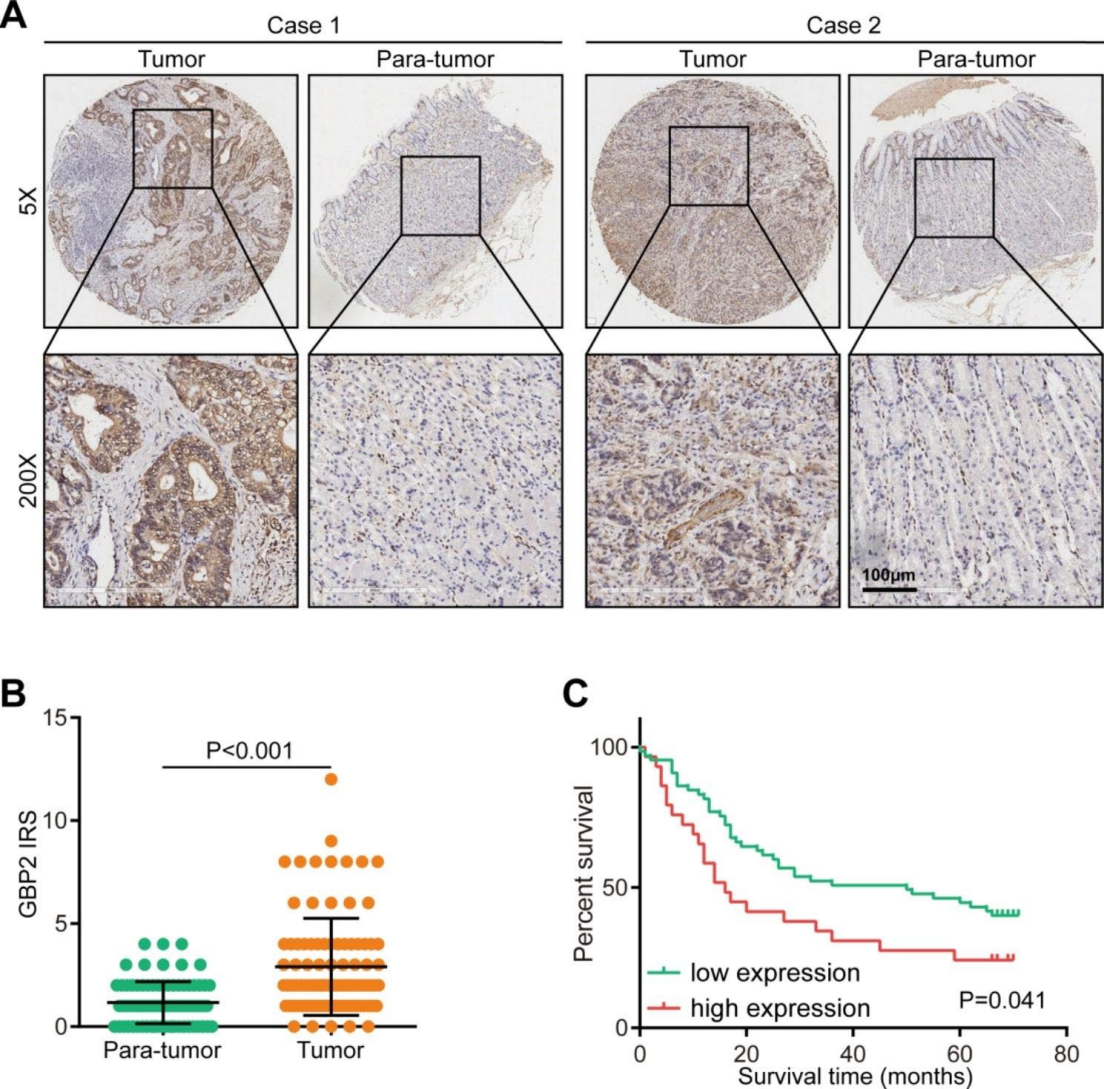
Expression and prognostic value of GBP2 in gastric cancer. **(A)** Representative images revealing GBP2 expression in tumor and para-tumor tissues using IHC staining. Magnification, 200×. **(B)** Semi-quantitative analysis of expression levels of GBP2 in tumor and para-tumor tissues. **(C)** Kaplan-Meier analysis showing overall survival (OS) of patients with low or high GBP2 expression in gastric cancer

First of all, we examined the expression of GBP2 in gastric cancer. In the GEPIA database, GBP2 was significantly upregulated in tumor tissues compared with para-tumor tissues (Figure S1A). To further confirm the above finding, we also conducted IHC statin in gastric cancer TMA. The results showed that GBP2 protein was located in cytoplasm and highly expressed in tumor tissues (Fig. 1A-B). Then, the prognostic value of GBP2 was also checked in public and in-house cohorts. The results showed that patients with high GBP2 expression exhibited poorer prognosis compared with those with low expression in the Kaplan-Meier plotter (Figure S1B). In addition, in the in-house cohort, GBP2 expression was also associated with unfavorable clinical outcome (Fig. 1C). Overall, GBP2 is significantly upregulated in gastric cancer and correlated with poor prognosis.

### Enrichment analysis of GBP2 in gastric cancer

**Fig. 2:**
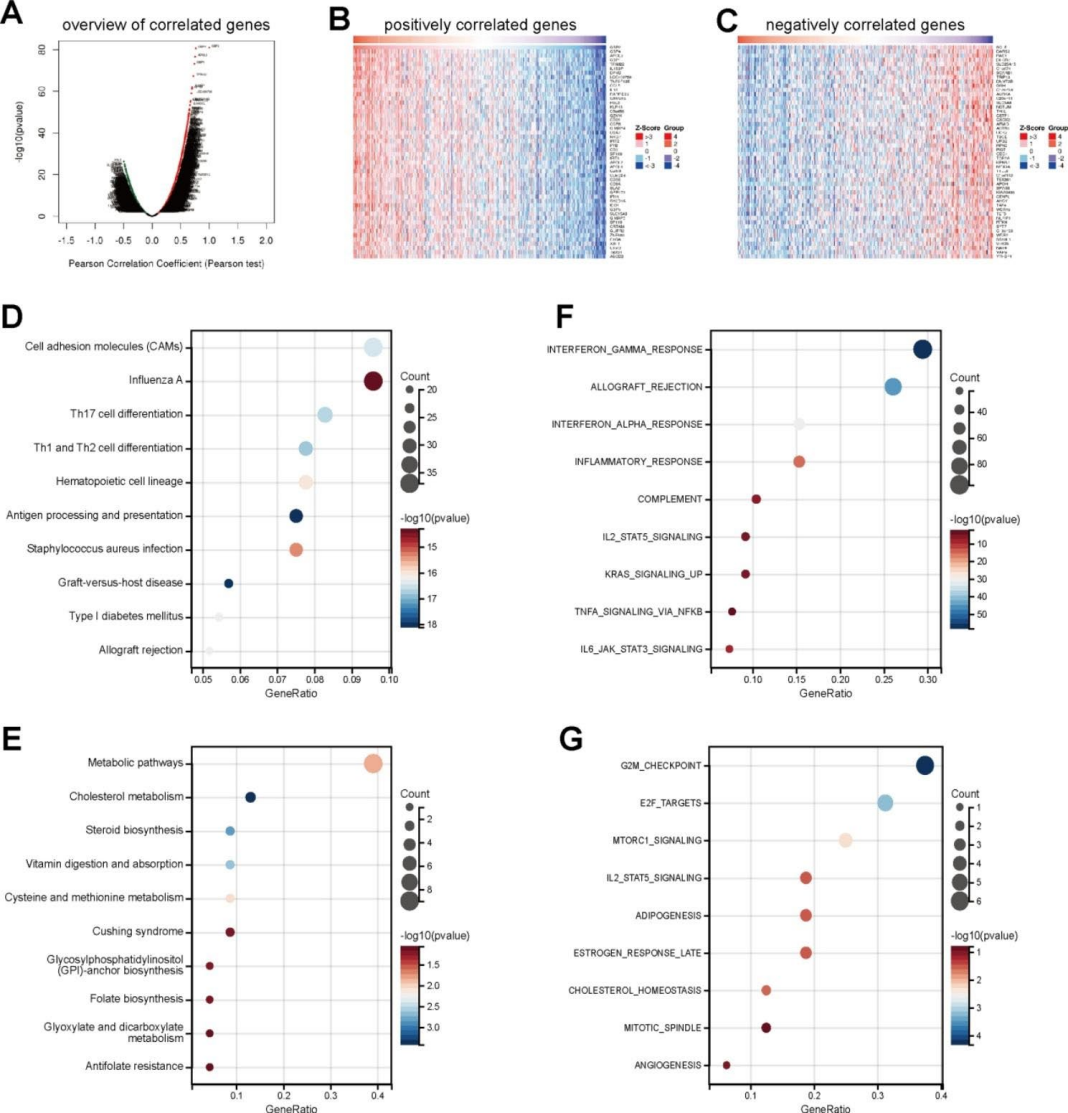
Co-expressed genes and enrichment analysis of GBP2 in gastric cancer. **(A)** The global GBP2 highly associated genes identified by Pearson test in gastric cancer cohort. **(B)** Heat maps showing top 50 genes positively associated with gastric cancer in HCC. **(C)** Heat maps showing top 50 genes negatively associated with gastric cancer in HCC. **(D)** Enrichment analysis of the positively correlated genes in the term of KEGG analysis. **(E)** Enrichment analysis of the negatively correlated genes in the term of KEGG analysis. **(F)** Enrichment analysis of the positively correlated genes in the term of Hallmark analysis. **(G)** Enrichment analysis of the negatively correlated genes in the term of Hallmark analysis

To obtain comprehensive insights into the biological functions of GBP2 in gastric cancer, the Linked Omics was used to seek the genes co-expressed with GBP2 in TCGA cohort. Genes significantly associated with GBP2 were exhibited in Figure ​Figure 2 A. The top 50 significant genes positively and negatively associated with GBP2 were shown in the heat map (Fig. 2B-C). In the term of KEGG analysis, the positively correlated genes were enriched in cell adhesion molecules, influenza A, Th17 cell differentiation, and so on, the negatively positively correlated genes were enriched in metabolic pathways, cholesterol metabolism, steroid biosynthesis, and so on (Fig. 2D-E). In the term of Hallmark analysis, the positively correlated genes were enriched in interferon-γ response, allograft rejection, interferon-α response, and so on, the negatively positively correlated genes were enriched in G2M_checkpoint, E2F_targets, MTORC1_signaling, and so on (Fig. 2F-G). Taken together, GBP2 is positively associated with immune response in gastric cancer.

### GBP2 was related to an inflamed tumor microenvironment in gastric cancer

**Fig. 3:**
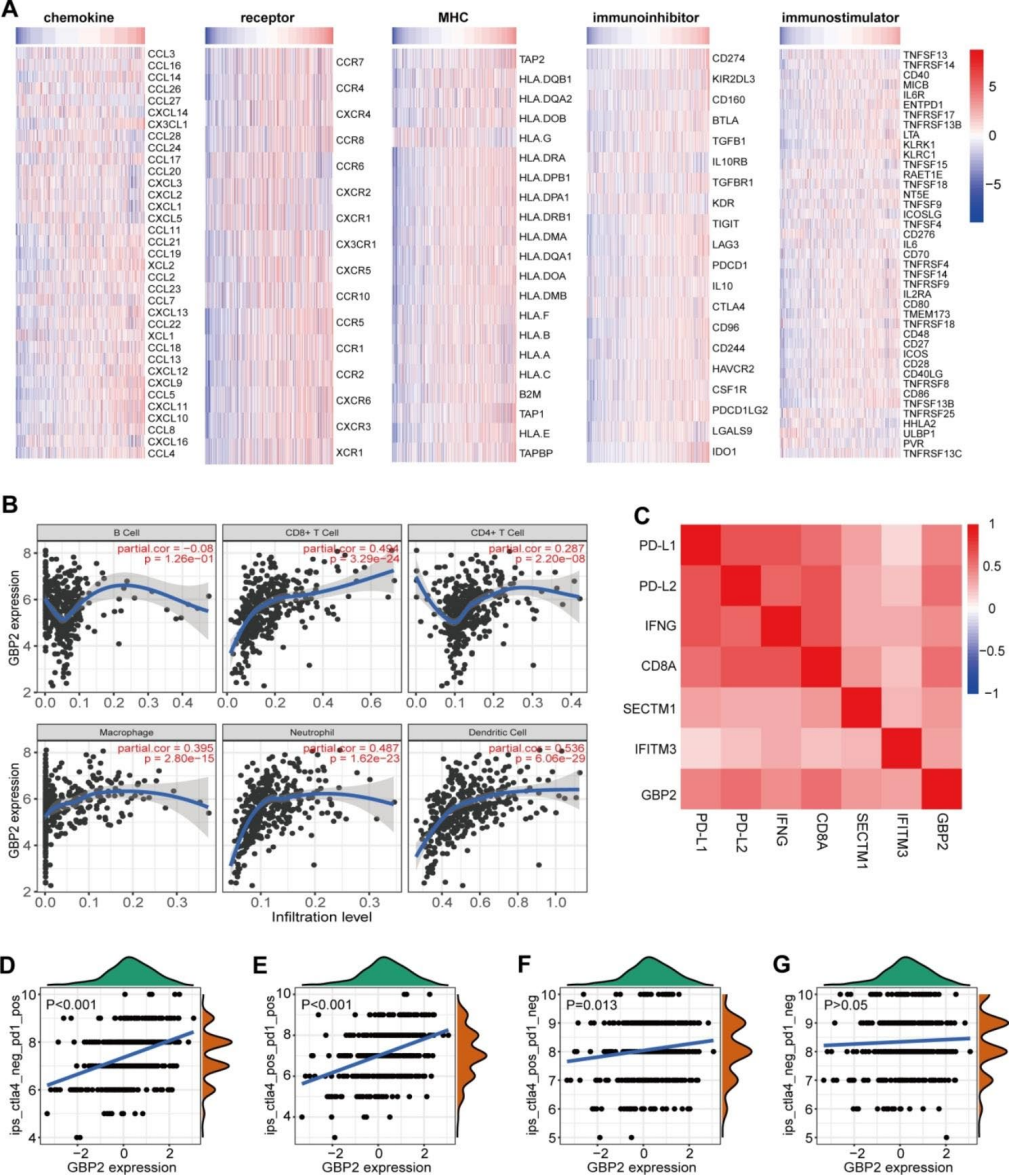
The correlations between GBP2 expression and TIME features in gastric cancer. **(A)** Correlations between GBP2 and immunomodulators expression, including chemokines, receptors, MHCs, immunoinhibitors, and immunostimulators. **(B)** Correlations between GBP2 and TIICs levels estimated by the TIMER tool. **(C)** Correlations between GBP2 and immunotherapy biomarkers, including PD-L1, PD-L2, IFNG, CD8A, SECTM1, and IFITM3. **(D-G)** Correlations between GBP2 and four IPS scores

Considering that GBP2 was associated with multiple immune-related processes, we next explored the precise immunological role of GBP2 in gastric cancer. A majority of chemokines, receptors, major histocompatibility complex (MHC) molecules, immunoinhibitors, and immunostimulators were notably correlated with GBP2 in gastric cancer (Fig. 3A). The expression of GBP2 was positively related to the infiltration levels of most immune cells estimated by the TIMER algorithm, especially CD8^+^ T cells, dendritic cells, and neutrophils (Fig. 3B). In addition, the expressions of immunotherapy biomarkers, such as PD-L1, PD-L2, IFN-γ, CD8A, SECTM1, and IFITM3 [32, 33], were also positively correlated with GBP2 expression (Fig. 3C). Moreover, GBP2 was positively related to IPS score as well (Fig. 3D-G). To sum up, all findings suggest that GBP2 is related to an inflamed TIME in gastric cancer, and may be a potential biomarker for immunotherapy.

### GBP2 predicted immunotherapeutic responses in gastric cancer

**Fig. 4:**
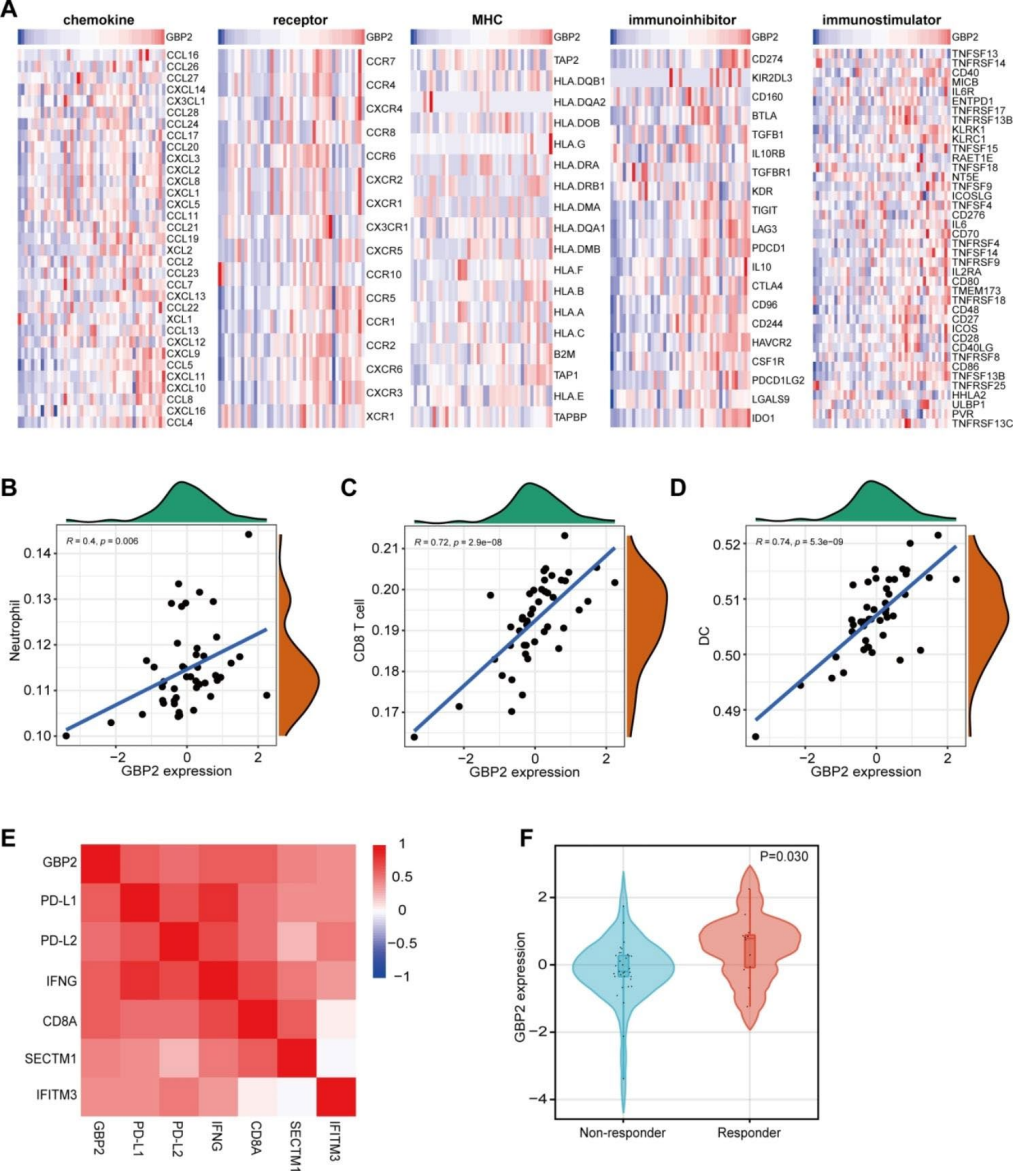
The value of GBP2 in predicting immunotherapeutic responses in gastric cancer. **(A)** Correlations between GBP2 and immunomodulators expression, including chemokines, receptors, MHCs, immunoinhibitors, and immunostimulators. **(B-D)** Correlations between GBP2 and neutrophils, CD8^+^ T cells, & dendritic cells estimated by the TIMER tool. **(E)** Correlations between GBP2 and immunotherapy biomarkers, including PD-L1, PD-L2, IFNG, CD8A, SECTM1, and IFITM3. **(F)** Expression of GBP2 in tumors with different immunotherapeutic responses

**Fig. 5:**
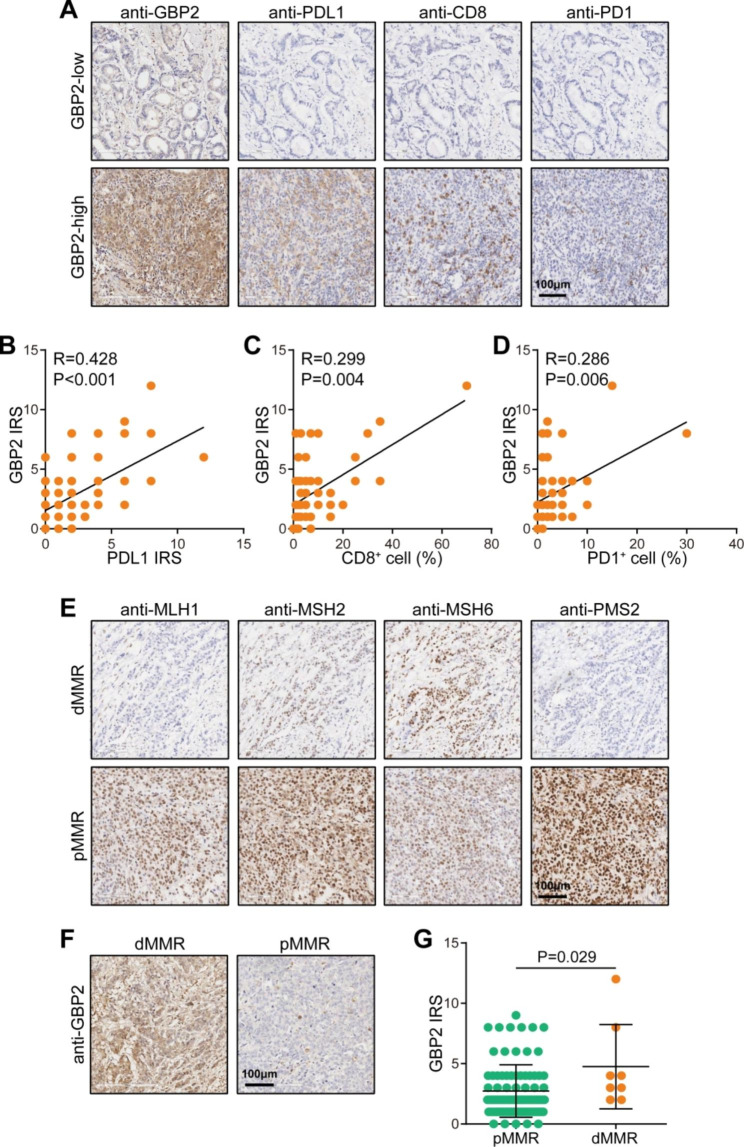
Validation of GBP2 and TIME features in gastric cancer. **(A)** Representative images revealing PDL1, CD8, and PD1 in tumor tissues with low and high GBP2 expression using IHC staining. Magnification, 200×. **(B)** Correlation between GBP2 and PDL1 expression. **(C)** Correlation between GBP2 and CD8^+^ cell levels. **(D)** Correlation between GBP2 and PD1^+^ cell levels. **(E)** Representative images revealing tumors with dMMR and pMMR status using IHC staining. Magnification, 200×. **(F)** Representative images revealing GBP2 expression in tumors with dMMR and pMMR status using IHC staining. Magnification, 200×. **(G)** Semi-quantitative analysis of expression levels of GBP2 in tumors with dMMR and pMMR status

To further validate the immune-correlation of GBP2 in gastric cancer. An immunotherapy cohort was used. Similar to the findings in the TCGA cohort, GBP2 was positively associated with most chemokines, receptors, major histocompatibility complex (MHC) molecules, immunoinhibitors, and immunostimulators (Fig. 4A). In addition, GBP2 was positively correlated with CD8^+^ T cells, dendritic cells, and neutrophils, but not obviously correlated with B cells, CD4^+^ T cells and macrophages (Fig. 4B-D, Figure S2A-C). Moreover, GBP2 was also positively related to the expressions of immunotherapy biomarkers (Fig. 4E). Furthermore, GBP2 was negatively correlated with four MMR gene expressions (Figure S3). More immediately, GBP2 was overexpressed in gastric cancer tumors with the well immunotherapeutic response (Fig. 4F). To validate the correlation between GBP2 and established immunotherapy biomarkers, we conducted the IHC analysis on the in-house cohort. The results showed GBP2 protein expression was positively correlated with PD-L1 expression, CD8^+^ cell abundance, and PD1^+^ cell abundance (Fig. 5A-D). Moreover, we detected MMR proteins using paraffin samples and checked the association between GBP2 and MMR status. The results showed that GBP2 was significantly highly expressed in dMMR samples (Fig. 5E-G). Totally, GBP2 is positively correlated with immunotherapeutic biomarkers and responses in gastric cancer.

### Pan-cancer analysis of the immune-correlation of GBP2

**Fig. 6:**
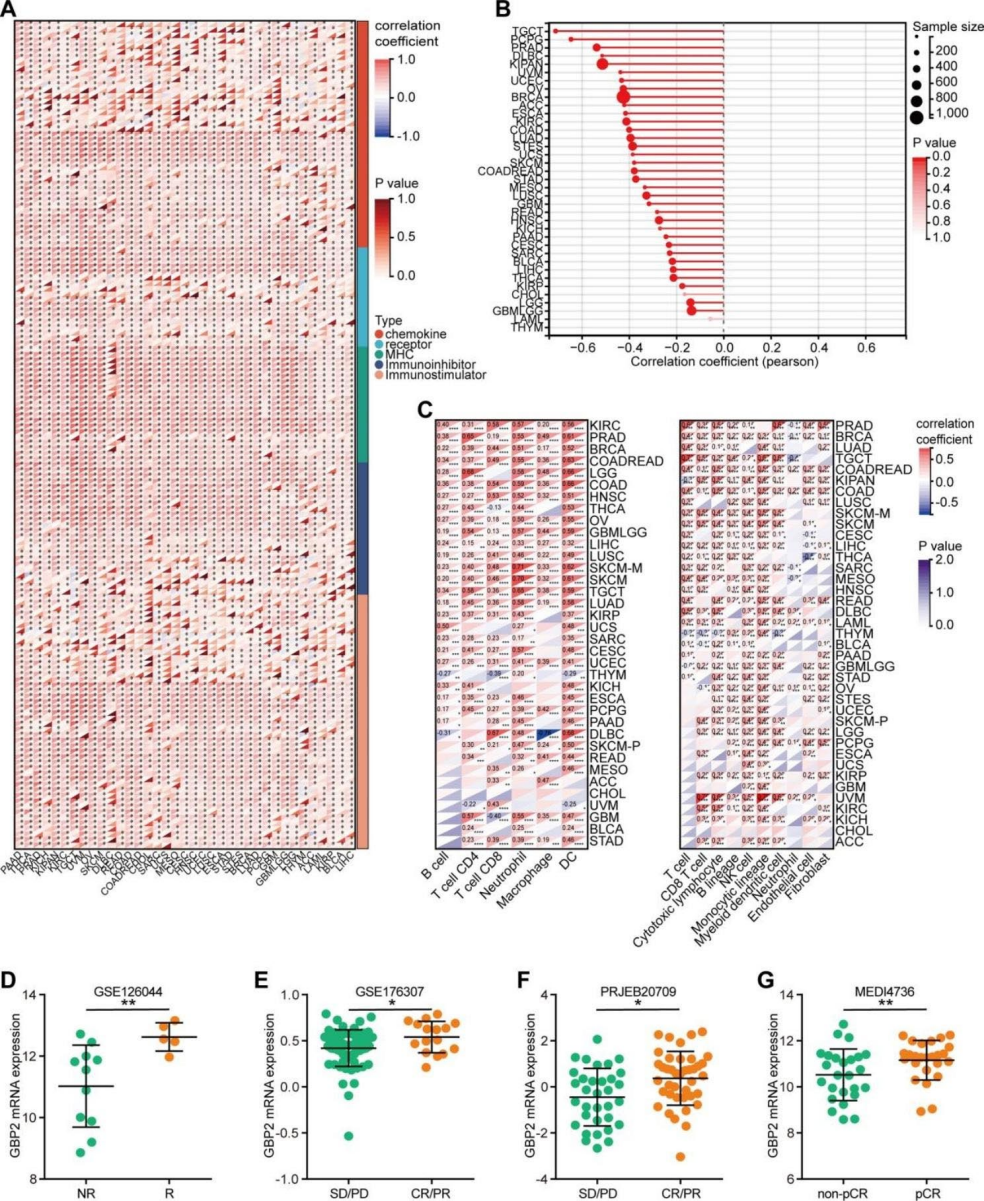
Pan-cancer analysis of the immuno-correlation of GBP2. **(A)** Correlations between GBP2 and 150 immunomodulators (MHC, receptors, chemokines, immunoinhibitors, and immunostimulators) in pan-cancer. **(B)** Correlations between GBP2 and tumor purity in pan-cancer. **(C)** Correlations between GBP2 and TIICs estimated by TIMER and EPIC algorithms in pan-cancer. **(D-G)** Expression of GBP2 in tumors with different immunotherapeutic responses in GSE126044 (non-small cell lung cancer), GSE176307 (urothelial carcinoma), PRJEB23709 (melanoma), and MEDI4736 (breast cancer) cohorts

Our results showed that GBP2 could detect immuno-hot tumors in gastric cancer and was connected to an inflamed TIME. GBP2’s immunological function in relation to other cancer types was unclear, nevertheless. The relationships between GBP2 and chemokines, receptors, MHC, immunoinhibitors, and immunostimulators were then examined. GBP2 was favorably linked with the expression levels of various immunomodulators, with the exception of a few cancer types (Fig. 6A). Moreover, in most cancer types, GBP2 was favorably connected with TIIC levels but negatively correlated with tumor purity (Fig. 6B-C). Also, in the cohorts of GSE126044 (non-small cell lung cancer), GSE176307 (urothelial carcinoma), PRJEB23709 (melanoma), and MEDI4736 (breast cancer), GBP2 was substantially expressed in tumors with good immunotherapeutic response (Fig. 6D-G). In addition, high GBP2 was associated with favorable prognosis in the term of both overall survival and progression-free survival (PFS) in the PRJEB23709 cohort (Figure S4A-B). Together, the information points to GBP2 as an immunotherapy pan-cancer biomarker, with the exception of a few tumor types.

## Discussion

Gastric cancer is the most common digestive malignant tumor in the world, with an incidence of 1,000,000 new cases one year. The mortality of gastric cancer goes high due to patients often being diagnosed at late stage, ranking third among causes of cancer-related death worldwide [34]. Endoscopic resection is the mainly treatment for early gastric cancer, while surgical resection and adjuvant chemotherapy are also main measures to treat gastric cancer with advanced stages [35].

With increasing discovery in molecular mechanisms of gastric cancer, immune escape is considered as one of the significant features of gastric cancer. Thus, immunotherapy now steps into the spotlight as part of comprehensive therapy [34]. The histological classification of gastric cancer is traditionally based on hallmarks of the epithelial tumor. However, other histological features exist in TIME as well, for example, tumor infiltrating lymphocytes or tumor-stroma ratio. These features will act as latent clinical prognostic factors in the future [36–38]. At present, the first-line biological agents for the treatment of gastric cancer include the HER2 antibodies, the anti-PD1 antibodies nivolumab and pembrolizumab, the anti-PD-L1 antibodies atezolizumab, avelumab and durvalumab [39]. It is hypothesized that immunotherapy combined with chemotherapy can improve the prognosis of gastric cancer. Cytotoxic chemotherapy may reconstruct TIME and promote immune-mediated anti-tumor effects, meanwhile PD-1 blockade together with the restoration of anti-tumor T cell response also enhance immunogenicity [40, 41].

The GBPs, originally isolated and identified from mouse cells, belong to the dynamin superfamily of large GTPases, which is mainly known for its multiple functions against invading microorganisms and pathogens. The GBP family is predominantly stimulated by IFN-γ, but other inflammatory cytokines such as IL-1α, IL-1β and TNF-α can also induce its expression in epithelial cells [42, 43]. The GBP family was discovered to be concerned in various signaling cascades [44]. Both p50 and NF-κB can enhance the promoter activity of GBP5. IAV infection induced GBP5 expression through NF-κB/p50 signaling, and GBP5 also stimulated IFNs, for example IFN-β and IFN-γ, and other downstream cytokine production to hamper viral replication in turn [45]. Moreover, at the early stage of Kaposi’s sarcoma-associated herpesvirus infection, GBP1 was found upregulated via NF-κB pathway [46].

Different from the protective roles of GBPs in fighting against microorganisms, its biological functions in tumors are not well characterized to a large extent. Xu et al. illustrated that GBP3 was overexpressed in glioblastoma and promoted tumor proliferation via activating the p62-ERK1/2 axis, and high GBP3 level diminished the sensitivity of glioblastoma to temozolomide treatment by enhancing DNA damage repair [47]. Yu et al. found that the expression of GBP2 was highly upregulated in glioblastoma multiforme, enhancing tumor invasion via Stat3/fibronectin pathway, which may predict poor prognosis for patients [48]. However, there is still no definite evidence that GBP2 is related to immunotherapy in gastric cancer.

In this research, we conducted comprehensive analysis of the immuno-correlation of GBP2 in gastric cancer. We found that GBP2 was highly expressed in gastric cancer and associated with poor prognosis. In addition, GBP2 was associated with immune-hot TIME in gastric cancer. Wang et al. revealed the high correlation among GBP2, high CD8^+^ T cell infiltration and better efficacy of PD-1 blockade response in colorectal cancer. They found that low expression of GBP2 was associated with weakened immune responses and poor prognosis of colorectal cancer patients, which suggested that GBP2 could serve as a potential immunotherapy target for colorectal cancer [14]. Similarly, GBP2 was also correlated with multiple immunotherapy biomarkers and the well response to immunotherapy in gastric cancer. In renal cell carcinoma, a serial of studies tried to explain the potential mechanisms. GBP2 could regulate PD‑L1 expression via STAT1 signaling [49]. Given that GBP2 could mediate STAT1 signaling in both colorectal cancer and renal cell carcinoma [14, 49], we supposed that GBP2-STAT1 axis might be critical for regulating the tumor immune status in a variety of malignancies. However, the supposition should be further validated.

## Conclusions

This study uncovers that GBP2 expression is associated with the immuno-hot TMIE in gastric cancer and could predict the immunotherapeutic responses. The pan-cancer study also shows that GBP2 is a marker for high immunogenicity in the majority of tumor types. All things considered, GBP2 may be a novel biomarker for determining tumor immunogenicity and directing immunotherapy.

### Electronic supplementary material

Below is the link to the electronic supplementary material.

Supplementary Material 1

## Data Availability

Data and materials can be provided upon reasonable request to the corresponding author. The datasets generated and/or analyzed during the current study are available in the UCSC Xena website (The pan-cancer (TOIL RSEM tpm) and gastric cancer (IlluminaHiSeq) RNA-sequencing data as well as clinical annotations), the GEO and TIDE databases (PRJEB25780, PRJEB23709, GSE176307, GSE126044, and MEDI4736).
