# Supplementary material for: GBP2 is a prognostic biomarker and associated with immunotherapeutic responses in gastric cancer
Source: BMC Cancer. 2023 Oct 2;23:925. doi: 10.1186/s12885-023-11308-0 (PMC10544588; doi:10.1186/s12885-023-11308-0)
Supplement: Supplementary file 1 — Supplementary Material 1 [file 12885_2023_11308_MOESM1_ESM.docx]

**Supplementary Figures**


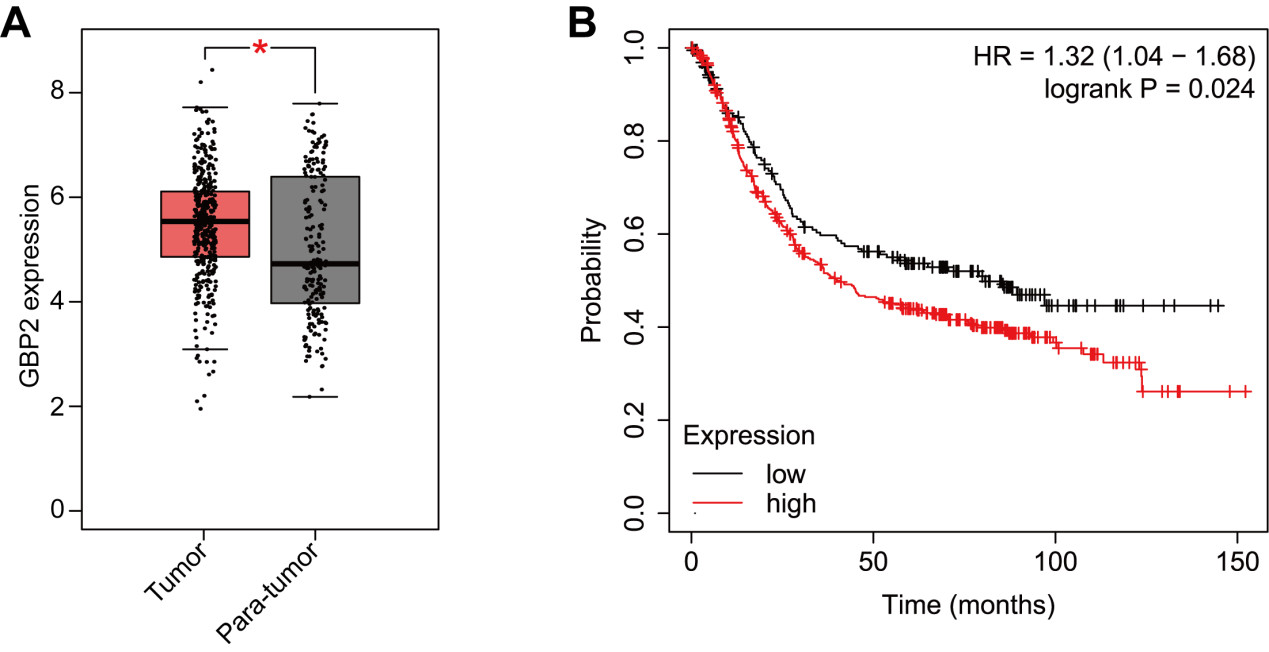


**Figure S1. Expression and prognostic value of GBP2 in gastric cancer (extend).** (A) Expression of GBP2 in tumor and para-tumor tissues in the GEPIA database. (B) Kaplan-Meier analysis showing OS of patients with low or high GBP2 expression in gastric cancer in the [Kaplan-Meier plotter](http://www.baidu.com/link?url=XGP4liI_Ks1AswbAaZ9FaDFh7mPAYZRUK48sjdslQI83ixjzL50i904i0O9heb00Mdd8pnhw0-UZwVLY0lSnMa) database.

**
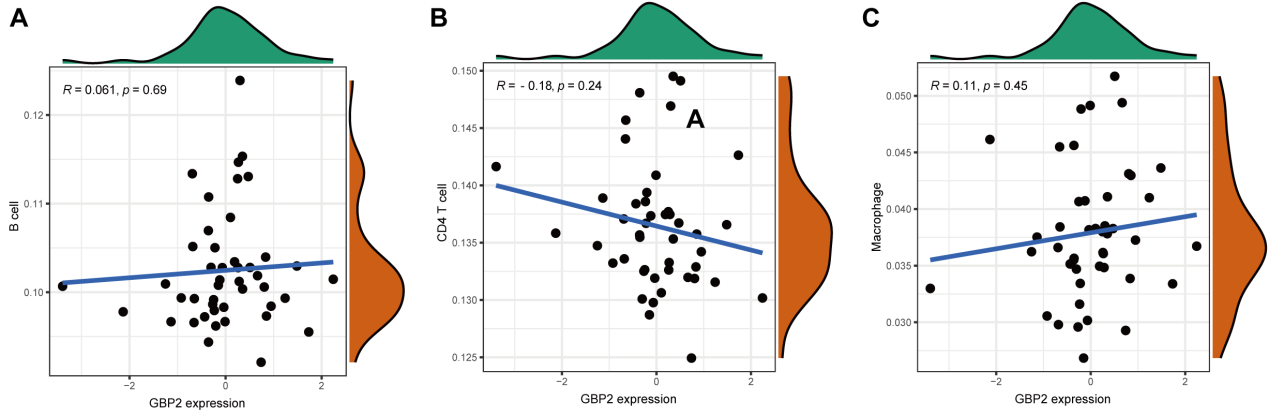
**

**Figure S2. Correlations between GBP2 and (A) B cells (B) CD4^+^ T cells, as well as (C) macrophages estimated by the TIMER tool.**

**
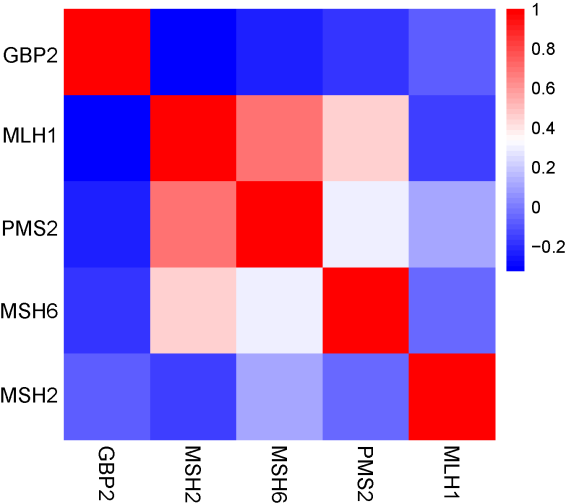
**

**Figure S3. Correlations between GBP2 and DNA repair genes expressions.**

**
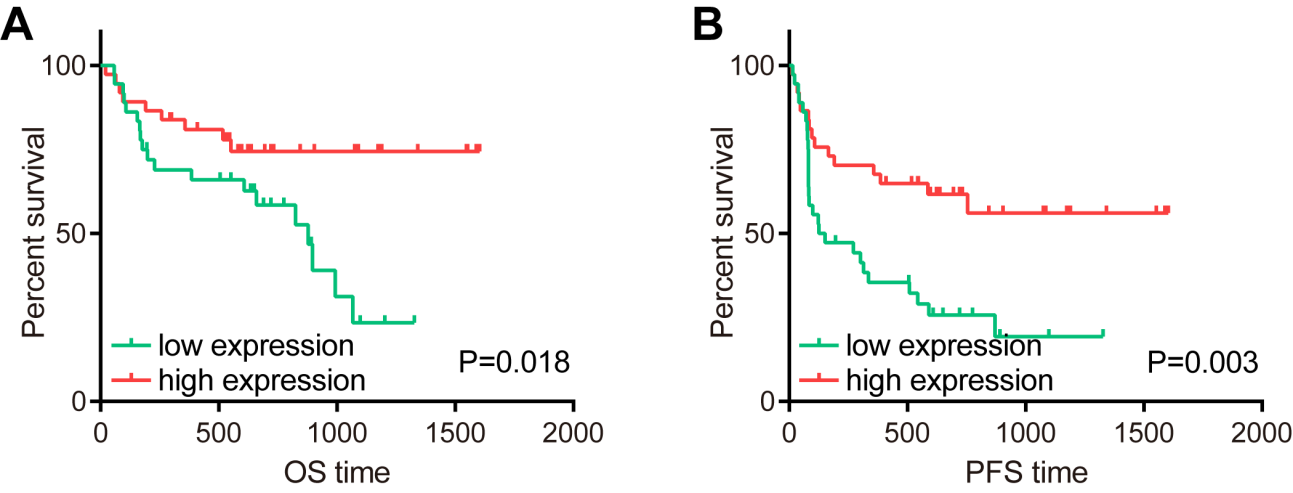
**

**Figure S4. Kaplan-Meier analysis of patients with low or high GBP2 expression in the PRJEB23709 cohort.** (A) Overall survival (OS). (B) Progression-free survival (PFS).

**Supplementary Tables**

**Table S1. Summary of abbreviations in the TCGA dataset.**

| Abbreviation | Full name |
| --- | --- |
| ACC | adrenocortical carcinoma |
| BLCA | bladder urothelial carcinoma |
| BRCA | breast invasive carcinoma |
| CESC | cervical & endocervical cancer |
| CHOL | cholangio carcinoma |
| COAD | colon adenocarcinoma |
| DLBC | lymphoid neoplasm diffuse large B-cell lymphoma |
| ESCA | esophageal carcinoma |
| GBM | glioblastoma multiforme |
| HNSC | head and neck squamous cell carcinoma |
| KICH | kidney chromophobe carcinoma |
| KIRC | kidney renal clear cell carcinoma |
| KIRP | kidney renal papillary cell carcinoma |
| LAML | acute myeloid leukemia |
| LGG | brain lower grade glioma |
| LIHC | liver hepatocellular carcinoma |
| LUAD | lung adenocarcinoma |
| LUSC | lung squamous cell carcinoma |
| MESO | mesothelioma |
| OV | ovarian serous cystadenocarcinoma |
| PAAD | pancreatic adenocarcinoma |
| PCPG | pheochromocytoma and paraganglioma |
| PRAD | prostate adenocarcinoma |
| READ | rectum adenocarcinoma |
| SARC | sarcoma |
| SKCM | skin cutaneous melanoma |
| STAD | stomach adenocarcinoma |
| TGCT | testicular germ cell tumors |
| THCA | thyroid carcinoma |
| THYM | thymoma |
| UCEC | uterine corpus endometrial carcinoma |
| UCS | uterine carcinosarcoma |
| UVM | uveal melanoma |
